# Moral Distress and Its Determinants among Nursing Students in an Italian University: A Cross-Sectional Study

**DOI:** 10.3390/nursrep14030160

**Published:** 2024-08-27

**Authors:** Giampiera Bulfone, Valentina Bressan, Irene Zerilli, Antonio Vinci, Rocco Mazzotta, Fabio Ingravalle, Massimo Maurici

**Affiliations:** 1 Department of Medical, Surgical Science, and Advanced Technology “GF Ingrassia”, University of Catania, 95123 Catania, Italy; 2 Department of Specialistic Medicine, Teaching University Hospital of Udine, 33100 Udine, Italy; 3 Doctoral School in Nursing Sciences and Public Health, University of Rome “Tor Vergata”, 00133 Rome, Italy; 4 Department of Biomedicine and Prevention, University of Rome “Tor Vergata”, 00133 Rome, Italy; rocco.mazzotta@uniroma2.it (R.M.);

**Keywords:** nursing, moral distress, nursing students, cross-sectional

## Abstract

Background: Moral Distress (MD) is a unique form of distress that occurs when people believe they know the ethically correct action to take but are constrained from doing so. Limited clinical experience and insufficient ethical knowledge contribute to nursing students’ MD, which can potentially cause negative outcomes. The aims of this study are: (1) to describe the MD intensity of nursing students, and (2) to analyze differences and associations between MD intensity and socio-demographic and academic variables. Methods: A cross-sectional study design with a convenience sample of the second, third, and delayed graduation students was included; only students willing to participate and who had attended their scheduled internships in the last six months were eligible for inclusion. To measure the level of MD, we used the It-ESMEE. We collected socio-demographic and academic variables. The data collection occurred from January 2024 to March 2024. Results: The students who adhered to the collection were N = 344. The findings reveal that the students perceived a high level of MD in situations related to clinical internship and class. They perceived higher levels of MD when nursing was not their first career choice, were separated or divorced, did not have children, and were not an employed student. The overall MD score is statistically significantly lower among students who had nursing as their first career choice (β = −0.267, *p* < 0.05), have children (β = −0.470, *p* < 0.01), and are employed (β = −0.417, *p* < 0.01). In contrast, being separated or divorced (β = 0.274, *p* < 0.01) was associated with a higher MD score. Conclusions: This study has some limitations: data reflect a local context, and the findings may not be generalizable to other regions or educational environments. Additionally, students’ recollections of their experiences could be influenced by the passage of time, and there may be a selection bias since only students willing to participate were included. The findings suggest that nursing education programs should incorporate more robust training in ethical decision-making and stress management to better prepare students for the moral challenges in their professional practice.

## 1. Introduction

### 1.1. Background and Rationale

Moral Distress (MD) is a unique form of distress that occurs when people believe they know the ethically correct action to take but are constrained from doing so; obstacles may lie in institutional structures and conflicts with co-workers [1]. Among students, it is caused by a situation in which the student perceives behaviors as morally inappropriate, disrespectful of subjects and their rights, and/or incompatible with the standards during academic training but cannot do anything about the situation due to a lack of experience or knowledge, situational constraints, hierarchical decision-making, lack of resources, or holding a subordinate position in the team [2]. MD is a particularly heinous form of distress, especially among healthcare professionals, because it threatens their core values and has ethical implications. It has been linked to burnout occurrence, job leaving, and poorer delivery of care towards patients [3,4]. MD presents a significant challenge in any healthcare environment, especially where ethical and moral complexities are prevalent [5,6]. As a result of their role and responsibilities, nurses often experience MD in clinical practice due to frequent encounters with situations that demand ethical and moral decision-making [7]. The causes and consequences of MD among nurses are well represented, and while various definitions are available, McCarthy and Gastmans describe MD as a broad concept encompassing the psychological and emotional suffering that occurs when individuals act in ways that conflict with their deeply held ethical values, principles, or moral commitments [8,9]. Jameton defined MD as the negative emotions that emerge when an individual recognizes the morally appropriate response to a situation but cannot act due to institutional or hierarchical constraints [10]. Conceptually, MD is understood as a feeling of powerlessness that stems from the inability to take an action perceived as ethically appropriate; it arises when individuals are unable to act in accordance with their judgment and values, leading them to view their moral involvement as inadequate [11].

In the nursing field, the Canadian Nurses Association (2008) expanded this definition, highlighting that MD can affect professionals’ values, commitments, and moral identity. Both practicing nurses and nursing students experience ethical dilemmas and MD. Although ethics courses are an integral part of undergraduate nursing curricula, nursing education programs often do not sufficiently prepare future professionals to face and manage ethical dilemmas and morally distressing situations [12]. Although nursing students are taught ethical theories and principles and how to address dilemmas, applying this knowledge can be difficult due to inter-professional and hierarchical conflicts, fear of repercussions, and insufficient guidance in clinical environments [13,14]. Limited clinical experience, low self-confidence, inadequate professional judgment, and insufficient ethical knowledge and training all contribute to nursing students’ inability to cope with MD [13,15]. This inability can ultimately diminish their moral sensitivity and capacity for moral courage, potentially leading them to enter the workplace already experiencing apathy and compassion fatigue. The consequences of MD in nursing students may include collective incivility of students, cheating and copying, bullying, communication problems with patients, and a pervasive feeling of impotence [8,16]. These and other effects of moral distress have been linked to a decline in quality of care and higher turnover rates [17]. Consequently, nursing students need the skills and abilities to effectively manage MD, and it is the responsibility of nursing educators to prepare future nurses by providing learning experiences that enable them to reflect on and address ethical dilemmas [12].

### 1.2. Study Objectives

While a lot of studies delve into professionals’ MD experience, few primary studies are focused on healthcare students, and even less among nurses. Most of the current literature is centered on qualitative and descriptive presentations, and there are few quantitative primary studies available, usually with tiny samples that make difficult to generalize on conclusions [18,19]. We decided to explore and analyze the experiences of MD in nursing students to inform educators and clinical instructors about the challenges students face in morally distressing situations. The findings of this study will assist in enhancing the ethical competencies of nursing students, equipping them with the necessary skills for their daily professional practice. The aims of this study are:

1. To describe the MD intensity of nursing students;
2. To analyze the differences in MD in socio-demographic and academic variables;
3. To identify the most significant domains within MD occurrence.

## 2. Materials and Methods

### 2.1. Study Design

A cross-sectional study design was adopted and is reported here in accordance with the STrengthening the Reporting of OBservational studies in Epidemiology (STROBE) for cross-sectional studies [20].

### 2.2. Setting

We investigated the bachelor’s degree program in nursing at an Italian university that enrolls up to 500 new students per year, with a total of 1500 students in the program.

### 2.3. Participants

A convenience sample of the second-year, third-year, and delayed graduation students were eligible. The sample included nursing students who:

(a) were enrolled in the studied program from the 2021–2022 academic year (or previous academic years, but were still attending still attending their bachelor’s course);
(b) had attended at least one internship (worth at least 10 European Credit Transfer System—ECTS) in the six months before data collection;
(c) were willing to participate in the study.

### 2.4. Measurement

The questionnaire collects sociodemographic (age, gender, marital status, parental status, and employment condition) and academic data (year of the program, nursing as a first career choice, previous university courses, the condition of passing all exams in relation to the year of the course, the mean Grade Point Average (0–30), and ECTS obtained at the time of data collection). In Italy, the nursing bachelor’s degree is a three year program, totaling 180 ECTS. Each year, students complete 60 ECTS through a combination of theory and internship. To measure the level of MD, we used the Italian Moral Distress Scale for Nursing Students scale (It-ESMEE), validated by Mazzotta et al. among Italian nursing students [21]. The reason for choosing this scale is that it was translated and culturally adapted for nursing students in the Italian context. The It-ESMEE has five dimensions (Disrespect for the ethical dimension of vocational training, Authoritarian teaching practices, Lack of competence of the teacher, Improper institutional conditions to teach user care, Commitment of ethical dimension of user care), and a second-order factor for the overall It-ESMEE score for intensity and frequency. The first three dimensions (Disrespect for the Ethical Dimension of Vocational Training, Authoritarian Teaching Practices, and Lack of Teacher Competence) are related to the assessment of MD concerning classroom activities and the competence perceived by the student in the teacher. In contrast, the remaining two dimensions (Improper Institutional Conditions for Teaching Patient Care and Commitment to the Ethical Dimension of Patient Care) are related to internship activities and the students’ relationships with the healthcare team. This scale was derived by translation and cultural adaptation of the original ESMEE developed by Bordignon et al. [22]. The It-ESMEE consisted of 31 items relating to MD assessment in academic environments (with teachers), organizational environments, and clinical practice. Each item had two scales: one for MD intensity and one for frequency, using a 7-point Likert-type scale (ranging from 0 = “no distress” to 6 = “severe distress”). High scores indicated high levels of MD intensity and frequency [21]. For this study, we modified the assessment of frequency to a two-point Likert scale (0 = no exposure to the situation, 1 = exposure to the situation), and included in the analysis only those students who reported exposure to the situation.

### 2.5. Data Collection Procedures and Ethical Issues

Data collection occurred from January 2024 to March 2024 through a series of structured online surveys using a Google Form. The surveys were collected during live meetings after university lessons, where two researchers (GB and VB) explained the aim of the survey. They also provided the students with instructions for completing the MD assessment and clarified that intensity assessment was required for each item. For frequency, students indicated if they were exposed to the specific situation (0 = not exposed; 1 = exposed). During the data collection period, the researchers were available to address any students’ clarification request.

### 2.6. Statistical Analysis

Statistical analyses were performed using SPSS 26 (IBM Corp. Armonk, NY, USA). The socio-demographic and academic characteristics of the students were analyzed using frequencies and percentages for categorical variables, and means and standard deviations (SD, ±), and also Medians and Inter-Quartile Range (IQR), for continuous variables. To report the global frequency of exposure among nursing students, the frequency of studied events was simplified into a dichotomous form (0: not exposed; ≥1: exposed).

We excluded subjects who reported 0 on the frequency scale, as they had no direct experience with the situation described in the 31 It-ESMREE items. Univariable and multivariable analysis were conducted for all dimensions and for the overall result of It-ESMEE to examine possible predictors of the reported MD intensity. A univariate analysis assessed the effect of each variable individually, and those with a possibly significant effect (*p* ≤ 0.15) were included into the multivariate model.

To test for differences among categorical variables, we applied *t*-test or one-way analysis of variance (ANOVA) when appropriate. All the required assumptions for *t*-test and ANOVA were evaluated (no significant outliers, dependent variable normally distributed, and homogeneity of variance) [23]. A Bonferroni correction was applied to *p*-values for pairwise comparisons. Normal distribution was assessed through visual inspection of the Normal Q-Q plot.

## 3. Results

### 3.1. Participants Characteristics

Among the 1000 second-year, third-year, and delayed in graduation students, 57.6% (n = 576) were potentially eligible for inclusion; 346 answered the survey (60.06%), and 2 of them refused to give consent for data analysis and were therefore excluded. The final response rate was 59.7% (344).

The sample characteristics are indicated in Table 1. A total of 344 nursing students participated in the study. They mostly attended the second- (75.29%) and third-year (18.6%) of the program, while 6.1% were delayed in graduation. They had a mean age of 23.12 years (±5.3) with a range of 18–53 years. In total, 73.26% were female, 86.92% were single, 95.03% did not have children, 79.65% were employed students during the program, and 77.03% had attended previous university courses before the nursing program; 64.24% attended the nursing program as their first career choice, and 64.83% passed every exam of the previous year, with a mean Grade Point Average (GPA) of 25.43 (±1.49) and a mean of 70.27 (±24.58) ECTS attended.

### 3.2. Moral Distress Intensity

The situations in which the students experienced higher levels of MD were in Q7 (Perceiving divergences between the way clinical nurses and teachers perform procedures) and Q10 (Identifying users’ access difficulties to care, such as long waiting lists, no hospital beds, or long waits in the emergency room), with mean scores above 3.00. Other situations in which the students perceived high levels of MD, with scores ≥ 2.8, were in Q4 (Identifying a mismatch between theoretical knowledge and practical application in one’s teaching–learning process) and Q12 (Improvising to cope with a lack of material in user care). The lower score below 2.3 mean was in item Q2 (Perceive change of a fellow student’s grades, by the teacher, to avoid failure), Q16 (Observe improper user care provided by students), Q19 (Observe inappropriate information to the user—e.g., about therapeutic project, discharge), Q22 (Observe a breach of confidentiality/professional secret of the user’s personal information), and Q30 (Experience delegation of nursing care to users’ relatives—e.g., asking relatives to do hygienic care for nursing workload). The MD intensity levels for all items and their respective dimensions, as perceived by nursing students, are detailed in Supplementary Table S1. We removed the students who had not experienced the specific situation described in the item; for each item, we indicated the number of subjects who actually responded.

**Figure 1:**
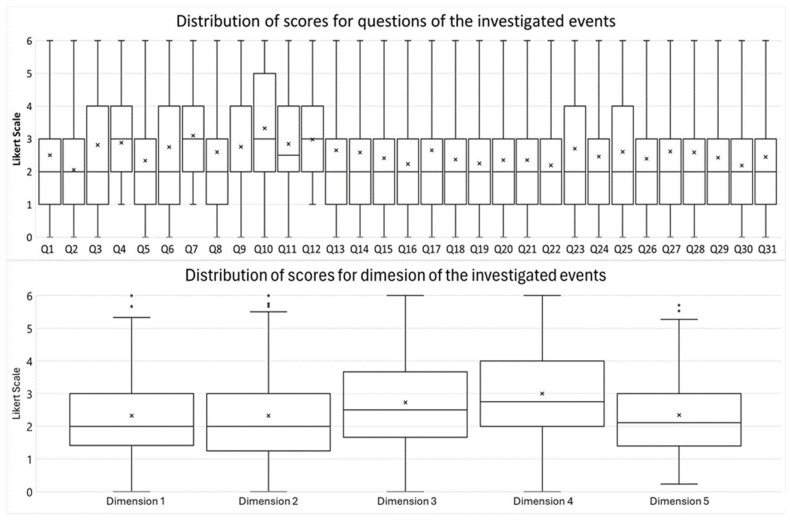
Distribution of scores for questions of the investigated events. The box plot illustrates the median, interquartile range (IQR), and minimum and maximum values. The mean score for each dimension is indicated by ‘×’. For each Question and Dimension definition, see Supplementary Table S1.

D4 (“Improper institutional condition to teach user care”) and D3 (“Lack of competence of the teacher”) had the highest mean scores, while the lowest score was reported in D5 (“Commitment of the ethical dimension of care”) (Figure 1).

### 3.3. Determinants of Moral Distress Intensity by Socio-Demographic and Academic Variables

In Table 2, we present the differences in MD intensity among nursing students based on socio-demographic and academic variables. MD intensity varies significantly among different groups of students.

Employment Status: students who are employed during their nursing program experience lower MD intensity compared to those who are not employed. This is reflected consistently across all dimensions and in the overall MD score.

Marital Status: significant differences were observed in the “Improper institutional conditions to teach user care” dimension. Single and separated or divorced students perceive higher MD intensity than their married counterparts or those with a partner.

Nursing as First Career Choice: Students who selected nursing as their first career choice report lower MD intensity in the dimensions of “Authoritarian teaching practice” and “Lack of competence of the teacher”, as well as in their overall MD score.

The overall MD score is statistically significantly lower among those students who had nursing as their first career choice (β = −0.267, *p* < 0.05), have children (β = −0.470, *p* < 0.01), and are employed students (β = −0.417, *p* < 0.01); in contrast, the condition of separated/divorced (β = 0.274, *p* < 0.01) was related to a higher MD. The same variables are associated to the “Disrespect for the ethical dimension of vocational training” dimension. The “Authoritarian teaching practice” dimension is significantly associated to the career choice (β = −0.437, *p* < 0.01), and to employment status (β = −0.473, *p* < 0.01). Other explored associations are shown in Table 3.

## 4. Discussion

### 4.1. Key Results

The findings indicate that students reported high levels of MD intensity in scenarios encountered during their internships and classroom activities, while reporting lower scores in dimensions related to patient safety and privacy. Statistically significant differences in MD intensity were found between students who were employed versus those who were not, those who selected nursing as their first career choice versus those who did not, and those who were partnered or married versus those who were single or divorced/separated. Moreover, some socio-demographic and academic variables are associated with MD intensity. The mean MD intensity ranged from 2.34 to 3.01 across all five dimensions. The dimensions of Improper institutional condition to teach user care and Lack of competence of the teacher had the highest scores, consistent with findings from Bordignon et al. [22].

Nursing students identified two main issues of moral impact: one related to their internship and one related to their classroom experience. During their internship, they perceived MD when patients faced difficulties in accessing appropriate care, there were a lack of materials to provide care, a lack of continuity in patient care, and discrepancies between the procedures performed by clinical nurses and those taught by teachers. Specifically, students felt a high level of MD regarding the difficulties patients face in accessing appropriate care, such as long waiting lists, lack of hospital beds, and extended waiting times in the emergency room. These challenges not only cause significant distress to patients but also hinder the overall effectiveness of the healthcare system. This is a national problem recognized at various levels of government and within healthcare institutions, as it carries difficulties in clinical patient management, hospital wards administration, and even the delivery of urgent care [24,25]. In response, the Italian government has implemented a recent law aimed at reducing waiting lists and improving the allocation of resources within the healthcare system [26].

The second issue pertains to classroom experience, where students faced challenges due to having a teacher lacking sufficient competence. Factor and Guzman reported that professional competence requires teachers to be proficient in both theoretical knowledge and clinical skills [27]. Nursing students consider professional competence of their teachers to be one of the most important characteristics [28]. In Italy, nursing teachers can be employed either with temporary contracts, requiring a Master of Science in Nursing (ANVUR, 2011), or as part of the nursing faculty. These professionals often have roles in administration, such as leadership or risk management, rather than working directly in clinical settings. It is important for nursing educators to have confidence in the clinical activities they teach and to remain engaged in clinical practice. They also need support to maintain their competence and effectiveness in both educational and clinical settings [29]. By ensuring that nursing educators are well-supported and confident, educational institutions can enhance the quality of clinical education, thereby better preparing nursing students for the complexities of clinical practice.

The lowest average score in our study pertains to “Commitment of the ethical dimension of care” (2.34), differently from Bordignon et al., in which the dimension with the lowest score was “Disrespect for the ethical dimension of vocational training” (1.87) [22]. This dimension refers to situations related to the patient privacy, safety, dignity, or to observe students that perform procedures merely to improve their skills. We believe that students perceive lower MD in these situations because they do not feel directly responsible for this situation.

Our study also identifies an absence of existing literature concerning the association between high levels of MD and specific socio-demographic factors such as marital status (e.g., being separated or divorced), having no children, and not being an employed student. The finding that students experiencing these conditions reported higher levels of MD suggests that these factors might contribute to the emotional and ethical challenges faced by nursing students. This may indicate that the presence of a supportive partner or family responsibilities could play a role in mitigating MD. A partner or children might provide emotional support or create a sense of stability that helps buffer the stress associated with clinical and academic demands. Conversely, the absence of these support systems might exacerbate feelings of MD.

In our study, nursing students perceived higher levels of MD when nursing was not their first career choice, were separated or divorced, did not have children, and were not employed students. This study reveals a notable gap in the literature regarding the association between MD and nursing as first career choice. Our findings suggest that students for whom nursing was not their first career choice experienced higher levels of MD. This may indicate a lack of awareness or understanding of the nursing role and the challenging contexts in which nurses operate.

### 4.2. Strengths and Limitations

Nursing students experienced MD during their clinical internships, and the findings of this study could be very useful for informing educators and clinical instructors. This, in turn, could enhance the ethical competencies of nursing students, equipping them with the necessary skills for their daily professional practice [30]. There is a paucity of research focusing on MD among nursing students, making this one of the few extant published studies in this domain.

This study has several limitations that should be acknowledged. First, data collection was conducted at an Italian university, reflecting a local context. Consequently, the findings are specific to this setting and may not be generalizable to other regions or educational environments. Second, the study included only students who had attended at least one internship within the six months preceding data collection. This criterion may have introduced recall bias, as students’ recollections of their experiences could be influenced by the passage of time. Additionally, the inclusion criteria may have further introduced selection bias, as only those students willing to participate were included, potentially skewing the results [31]. Furthermore, this is a cross-sectional study, which captures data at a single point in time. As such, it reflects specific conditions and emotive states of the students at the time of data collection, which may not represent their experiences or attitudes over a longer period. These factors limit the external validity of the study, and caution should be exercised when attempting to apply these findings to broader populations or different contexts.

### 4.3. Nursing Clinical Implications

Nursing is an ethical profession with attention paid to the values, rights, duties, and responsibilities of its professionals [32]. Any situation that nursing students perceived as morally inappropriate causes high level of suffering, resulting in high MD intensity [10]; a recent study by Timmins also found that daily practices, such as pain control or the decision to restrain patients, can be a source of MD when deficient professional ethics from healthcare professionals is sensed by the student [33]. MD must be treated as a systemic problem requiring organizational solutions; individual responses and personal virtues are, at best, mild answers to the occurrence of the phenomenon, as organization’s policies may be contributing to many morally distressing situations [34,35].

Given the high levels of MD reported by students in clinical and classroom settings, it is crucial for nursing education programs to integrate comprehensive ethical training into the curriculum. Educators should focus on developing students’ ability to navigate ethical dilemmas, emphasizing practical approaches to resolving conflicts between ethical obligations and institutional constraints. The educators should include counseling services and peer support networks to help mitigate the impact of personal stressors on academic performance and MD [8].

The finding that students who chose nursing as their first career option experience lower MD suggests that a strong professional identity may buffer against ethical stress. Educators should therefore emphasize career choice and professional identity formation early in the program. Activities such as mentoring, reflective practice, and early exposure to clinical settings may help reinforce students’ commitment to the profession and reduce MD [8].

The results highlight the need for stress management training as part of the nursing curriculum. Nursing educators should provide students with tools and strategies to manage stress effectively, including mindfulness practices, resilience training, and access to mental health resources. This can help students better cope with the ethical and emotional challenges of their clinical experiences. To address the ongoing challenge of MD, educators should consider implementing regular assessments of students’ MD levels throughout their education. This can help identify students who may be at risk of experiencing high MD and allow for timely interventions, such as providing additional support or adjusting learning environments.

Longitudinal studies on MD should be a priority in research to track the development and progression of MD over time, particularly as students transition from education to professional practice. This could provide deeper insights into the long-term effects of MD and the effectiveness of interventions.

The study suggests that certain factors, such as having nursing as a first career choice, are associated with lower MD. Further research is needed to explore these protective factors in more depth, including how they can be cultivated or strengthened in educational and professional settings.

Since the study reflects a specific local context, comparative studies in different regions or educational environments are necessary to determine the generalizability of the findings. Such research could identify context-specific factors influencing MD and inform more tailored interventions.

Research should focus on evaluating the effectiveness of various ethical training programs in reducing MD among nursing students. This includes assessing different pedagogical approaches, such as simulation-based learning or interdisciplinary ethics education, to determine what methods are most effective in preparing students for the ethical challenges of their careers.

The influence of socio-demographic variables, such as marital status and employment, on MD warrants further investigation. Research could explore how these variables interact with other factors, like cultural background, financial stress, and social support systems, to provide a more comprehensive understanding of their impact on MD. By addressing these implications in practice and research, healthcare educators and institutions can better support nursing students and professionals in managing MD, ultimately leading to improved patient care and staff well-being.

## 5. Conclusions

The study identifies that certain groups of nursing students are more prone to higher levels of MD, indicating a need for targeted interventions. Educators and clinical instructors should focus on providing additional support and resources to these groups to enhance their ethical competencies and emotional resilience. The findings suggest that nursing education programs should incorporate more robust training in ethical decision-making and stress management to better prepare students for the moral challenges they will encounter during their professional practice. Such enhancements could help mitigate the risk of apathy and compassion fatigue.

Future research should evaluate the effectiveness of interventions aimed at reducing MD among nursing students. Additionally, longitudinal studies could provide deeper insights into how MD develops and changes as students transition from academic settings to professional practice.

## Figures and Tables

**Table 1:** Sample characteristics (N = 344). GPA: Grade Points average. ECTS: European Credits Transfer System.

Variable		N (%) or Mean ± SD (Min–Max)
Program year	2nd year	259 (75.29%)
	3rd year	64 (18.6%)
	Delayed Graduation	21 (6.1%)
Age		23.12 ± 5.3 (18–53)
Gender	Male	92 (26.74%)
	Female	252 (73.26%)
Marital status	Single	299 (86.92%)
	Married/with partner	3 (0.87%)
	Separated/divorced	42 (12.21%)
Parental status	No children	330 (95.93%)
	1 child	6 (1.74%)
	2 children or more	7 (2.03%)
	3	1 (0.29%)
Employment status during program	Employed	274 (79.65%)
	Not employed	70 (20.35%)
Nursing as first career choice	Yes	221 (64.24%)
	No	123 (35.76%)
Previous University program	Attended	79 (22.97%)
	Not attended	265 (77.03%)
Passed all exams of previous year	Yes	223 (64.83%)
	No	121 (35.17%)
GPA		25.43 ± 1.49 (21–29.33)
ECTS		70.27 ± 24.58 (4–153)

**Table 2:** Moral Distress intensity by Socio-demographic and academic variables. For each Dimension definition, see Supplementary Table S1. Statistically significant *p*-values are in bold.

Variables		Dimension 1	*p*-Value	Dimension 2	*p*-Value	Dimension 3	*p*-Value	Dimension 4	*p*-Value	Dimension 5	*p*-Value	Overall Moral Distress	*p*-Value
Immatricolation Year	2nd year	2.48 ± 1.22	0.116	2.52 ± 1.33	0.353	2.75 ± 1.31	0.452	3.06 ± 1.37	0.312	2.39 ± 1.15	0.179	2.54 ± 1.07	0.285
	3rd year	2.37 ± 1.13		2.28 ± 1.17		2.77 ± 1.31		2.96 ± 1.38		2.2 ± 1.04		2.4 ± 1.04	
	Delayed in graduation	1.93 ± 0.87		2.29 ± 1.06		2.39 ± 0.94		2.61 ± 1.1		2.13 ± 1.02		2.21 ± 0.9	
Gender	Male	2.46 ± 1.2	0.341	2.42 ± 1.27	0.446	2.73 ± 1.25	0.964	3.05 ± 1.34	0.401	2.31 ± 1.07	0.405	2.48 ± 1.03	0.68
	Female	2.32 ± 1.16		2.55 ± 1.33		2.74 ± 1.4		2.91 ± 1.4		2.43 ± 1.27		2.53 ± 1.15	
Marital status	Single	2.41 ± 1.16	0.46	2.47 ± 1.3	0.216	2.72 ± 1.28	0.142	3 ± 1.34	**0.049**	2.31 ± 1.11	0.072	2.48 ± 1.04	0.084
	Married/with partner	1.5 ± 0.71		1.17 ± 0.29		1.39 ± 0.35		1.25 ± 0.25		1.45 ± 0.11		1.4 ± 0.16	
	Separated/divorced	2.53 ± 1.38		2.44 ± 1.23		2.89 ± 1.36		3.22 ± 1.41		2.65 ± 1.22		2.7 ± 1.14	
Parental status	No child	2.45 ± 1.19	0.157	2.48 ± 1.29	0.157	2.76 ± 1.29	0.144	3.05 ± 1.35	0.057	2.36 ± 1.13	0.196	2.52 ± 1.06	0.74
	1 child	1.7 ± 0.45		1.06 ± 0.13		1.57 ± 0.37		1.56 ± 0.43		1.21 ± 0.2		1.46 ± 0.36	
	2 children or more	1.57 ± 1.22		2.13 ± 1.39		2.29 ± 1.51		2.32 ± 1.32		2.06 ± 0.83		1.99 ± 0.7	
Employment Condition	Yes	2.01 ± 1.09	**0.003**	2 ± 1.19	**0.001**	2.36 ± 1.22	**0.008**	2.58 ± 1.21	**0.004**	2 ± 0.91	**0.005**	2.14 ± 0.88	**0.002**
	No	2.52 ± 1.19		2.57 ± 1.29		2.82 ± 1.29		3.11 ± 1.37		2.43 ± 1.16		2.58 ± 1.08	
Nursing as first career choice	Yes	2.33 ± 1.18	0.06	2.3 ± 1.23	**0.005**	2.62 ± 1.28	**0.026**	2.92 ± 1.38	0.92	2.26 ± 1.11	0.079	2.4 ± 1.05	**0.031**
	No	2.59 ± 1.2		2.73 ± 1.34		2.94 ± 1.29		3.18 ± 1.3		2.48 ± 1.14		2.66 ± 1.07	
Previous University program	Yes	2.41 ± 1.18	0.795	2.49 ± 1.28	0.43	2.78 ± 1.3	0.266	3.05 ± 1.35	0.408	2.37 ± 1.11	0.457	2.51 ± 1.04	0.512
	No	2.45 ± 1.24		2.35 ± 1.31		2.59 ± 1.26		2.9 ± 1.37		2.26 ± 1.19		2.43 ± 1.12	
Passed all exams of previous year	Yes	2.29 ± 1.02	0.129	2.37 ± 1.21	0.351	2.7 ± 1.3	0.699	2.86 ± 1.32	0.139	2.21 ± 1.03	0.122	2.38 ± 0.96	0.155
	No	2.5 ± 1.27		2.51 ± 1.33		2.75 ± 1.29		3.09 ± 1.37		2.41 ± 1.17		2.55 ± 1.11	

**Table 3:** Determinants of moral distress intensity in nursing students. GPA: Grade Points average. ECTS: European Credits Transfer System. For each Dimension definitions, see Table 2.

Variables	Dimension 1				Dimension 2				Dimension 3				Dimension 4				Dimension 5				Moral Distress (Overall)			
	Univariate		Multivariate		Univariate		Multivariate		Univariate		Multivariate		Univariate		Multivariate		Univariate		Multivariate		Univariate		Multivariate	
	β	*p*-Value	β	*p*-Value	β	*p*-Value	β	*p*-Value	β	*p*-Value	β	*p*-Value	β	*p*-Value	β	*p*-Value	β	*p*-Value	β	*p*-Value	β	*p*-Value	β	*p*-Value
Age (years)	0.007	0.58	0.006	0.49	0.004	0.77	0.001	0.98	0.014	0.28	0.013	0.31	0.004	0.78	0.003	0.81	0.015	0.19	0.013	0.27	0.01	0.37	0.007	0.5
Gender	−0.145	0.34	−0.127	0.41	0.126	0.45	0.172	0.3	0.007	0.96	0.032	0.85	−0.142	0.4	−0.106	0.54	0.116	0.4	0.153	0.28	0.054	0.68	0.097	0.46
First career choice	−0.258	0.06	−0.275	<0.05	−0.422	<0.01	−0.437	<0.01	−0.032	<0.05	−0.343	<0.05	−0.259	0.09	−0.268	0.08	−0.224	0.08	−0.222	0.08	−0.258	<0.05	−0.267	<0.05
Marital status	0.047	0.64	0.23	<0.05	−0.036	0.74	0.102	0.38	0.063	0.55	0.228	0.06	0.078	0.49	0.264	<0.05	0.155	0.1	0.311	<0.01	0.096	0.27	0.274	<0.01
Parental status	−0.453	<0.01	−0.597	<0.01	−0.263	0.22	−0.215	0.35	−0.352	0.08	−0.453	<0.05	−0.485	<0.05	−0.638	<0.01	−240	0.18	−0.376	0.05	−0.345	<0.05	−0.47	<0.01
Previous University program	0.041	0.79	0.093	0.56	−0.135	0.43	−0.02	0.91	−0.185	0.27	−0.147	0.39	−0.145	0.41	−0.114	0.52	−0.108	0.46	−0.02	0.89	−0.089	0.51	−0.024	0.86
Employment condition	−0.507	<0.01	−0.473	<0.01	−0.569	<0.001	−0.561	<0.01	−0.467	<0.01	−0.387	<0.05	−0.53	<0.01	−0.423	<0.05	−0.43	<0.01	−0.431	<0.01	−0.441	<0.01	−0.417	<0.01
Passed all exams of previous year	0.21	0.13	0.178	0.2	0.14	0.35	0.114	0.44	0.057	0.7	0.028	0.85	0.229	0.14	0.195	0.21	0.198	0.12	0.164	0.19	0.17	0.15	0.137	0.24
GPA	−0.022	0.63	−0.008	0.86	−0.044	0.38	−0.009	0.86	−0.011	0.82	0.014	0.77	−0.009	0.85	0.015	0.77	−0.08	0.06	−0.06	0.15	−0.044	0.26	−0.023	0.56
ECTS	0.001	0.99	−0.002	0.52	−0.003	0.29	−0.004	0.12	0.001	0.62	−0.001	0.98	0.002	0.5	0.001	0.94	0.001	0.85	−0.002	0.39	0.001	0.92	−0.02	0.43

## Data Availability

Data is contained within the article or Supplementary Materials.

## Supplementary Materials

The following supporting information can be downloaded at: https://www.mdpi.com/article/10.3390/nursrep14030160/s1, Table S1: Moral Distress intensity in nursing students.

## Public Involvement Statement

No public involvement in any aspect of this research.

## Guidelines and Standards Statement

This manuscript was drafted against the STrengthening the Reporting of OBservational studies in Epidemiology (STROBE) for epidemiological research.

## References

[1] Jameton A. (1993). Dilemmas of moral distress: Moral responsibility and nursing practice. AWHONNs Clin. Issues Perinat. Womens Health Nurs..

[2] Jameton A. (1984). Nursing Practice: The Ethical Issues.

[3] Topal S., Çaka S.Y., Öztürkler S., Gürbüz Y. (2024). Burnout inpediatric nurses: Examining the relationship between moral distress and missed nursing care. J. Pediatr. Nurs..

[4] Delgado-Ron J.A., Tiwana M.H., Murage A., Morgan R., Purewal S., Smith J. (2024). Moral distress, coping mechanisms, and turnover intent among healthcare providers in British Columbia: A race and gender-based analysis. BMC Health Serv. Res..

[5] Kovanci M.S., Atli Özbaş A. (2024). Moral resilience and intention to leave: Mediating effect of moral distress. Nurs. Ethics.

[6] Hoskins K., Grady C., Ulrich C. (2018). Ethics Education in Nursing: Instruction for Future Generations of Nurses. Online J. Issues Nurs..

[7] Ulrich C.M., Taylor C., Soeken K., O’Donnell P., Farrar A., Danis M., Grady C. (2010). Everyday ethics: Ethical issues and stress in nursing practice. J. Adv. Nurs..

[8] Wojtowicz B., Hagen B., Van Daalen-Smith C. (2014). No place to turn: Nursing students’ experiences of moral distress in mental health settings. Int. J. Ment. Health Nurs..

[9] McCarthy J., Gastmans C. (2015). Moral distress: A review of the argument-based nursing ethics literature. Nurs. Ethics.

[10] Jameton A. (2017). What Moral Distress in Nursing History Could Suggest about the Future of Health Care. AMA J. Ethics.

[11] Rennó H.M.S., Ramos F.R.S., Brito M.J.M. (2018). Moral distress of nursing undergraduates: Myth or reality?. Nurs. Ethics.

[12] Escolar-Chua R.L. (2018). Moral sensitivity, moral distress, and moral courage among baccalaureate Filipino nursing students. Nurs. Ethics.

[13] Bickhoff L., Sinclair P.M., Levett-Jones T. (2017). Moral courage in undergraduate nursing students: A literature review. Collegian.

[14] Dimitriadou M., Papastavrou E., Efstathiou G., Theodorou M. (2015). Baccalaureate nursing students’ perceptions of learning and supervision in the clinical environment. Nurs. Health Sci..

[15] Comrie R.W. (2012). An analysis of undergraduate and graduate student nurses’ moral sensitivity. Nurs. Ethics.

[16] Klocko M.N. (2014). Academic Dishonesty in Schools of Nursing: A Literature Review. J. Nurs. Educ..

[17] Krautscheid L., DeMeester D.A., Orton V., Smith A., Livingston C., McLennon S.M. (2017). Moral Distress and Associated Factors Among Baccalaureate Nursing Students: A Multisite Descriptive Study. Nurs. Educ. Perspect..

[18] Gonella S., Viottini E., Gastmans C., Tambone S., Conti A., Campagna S., Dimonte V. (2024). Lived experience of ethical challenges among undergraduate nursing students during their clinical learning. Nurs. Ethics.

[19] Heng T.J.T., Shorey S. (2023). Experiences of moral distress in nursing students—A qualitative systematic review. Nurse Educ. Today..

[20] Von Elm E., Altman D.G., Egger M., Pocock S.J., Gøtzsche P.C., Vandenbroucke J.P. (2008). The Strengthening the Reporting of Observational Studies in Epidemiology (STROBE) statement: Guidelines for reporting observational studies. J. Clin. Epidemiol..

[21] Mazzotta R., De Maria M., Bove D., Badolamenti S., Saraiva Bordignon S., Silveira L.C.J., Vellone E., Alvaro R., Bulfone G. (2022). Moral distress in nursing students: Cultural adaptation and validation study. Nurs. Ethics.

[22] Bordignon S.S., Lunardi V.L., Barlem E.L.D., Dalmolin G.D.L., Da Silveira R.S., Ramos F.R.S., Barlem J.G.T. (2019). Moral distress in undergraduate nursing students. Nurs. Ethics.

[23] Lix L.M., Keselman J.C., Keselman H.J. (1996). Consequences of Assumption Violations Revisited: A Quantitative Review of Alternatives to the One-Way Analysis of Variance “F” Test. Rev. Educ. Res..

[24] Vinci A., Furia G., Cammalleri V., Colamesta V., Chierchini P., Corrado O., Mammarella A., Ingravalle F., Bardhi D., Malerba R.M. (2024). Burden of delayed discharge on acute hospital medical wards: A retrospective ecological study in Rome, Italy. PLoS ONE.

[25] Furia G., Vinci A., Colamesta V., Papini P., Grossi A., Cammalleri V., Chierchini P., Maurici M., Damiani G., De Vito C. (2023). Appropriateness of frequent use of emergency departments: A retrospective analysis in Rome, Italy. Front. Public Health.

[26] Misure Urgenti per la Riduzione dei Tempi Delle Liste di Attesa Delle Prestazioni Sanitarie. Decreto-Legge, GU n.132 7 June 2024. https://www.normattiva.it/uri-res/N2Ls?urn:nir:stato:decreto-legge:2024-06-07;73!vig=.

[27] Factor E.M.R., De Guzman A.B. (2017). Explicating Filipino student nurses’ preferences of clinical instructors’ attributes: A conjoint analysis. Nurse Educ. Today.

[28] Labrague L.J., McEnroe-Petitte D.M., D’Souza M.S., Hammad K.S., Hayudini J.N.A. (2020). Nursing faculty teaching characteristics as perceived by nursing students: An integrative review. Scand. J. Caring Sci..

[29] Al-Rjoub S.F., Diener E., AL-Fayyadh S. (2022). Nurse administrators as the cause of moral distress among nurse educators: A qualitative research study. J. Prof. Nurs..

[30] Sasso L., Bagnasco A., Bianchi M., Bressan V., Carnevale F. (2016). Moral distress in undergraduate nursing students: A systematic review. Nurs. Ethics.

[31] Nohr E.A., Liew Z. (2018). How to investigate and adjust for selection bias in cohort studies. Acta Obs. Gynecol. Scand..

[32] Tahmasbi S., Alavi A. (2023). Ethical Sensitivity and Moral Self-concept of Nursing Students During Internship: Factors and Assessment. Health Spirit. Med. Ethics.

[33] Rebecca T. (2024). Student nurses experiences of moral distress: A concept analysis. J. Adv. Nurs..

[34] Usset T., Fantus S. (2023). Moral Distress Is a Systemic Problem Requiring Organizational Solutions. Am. J. Bioeth..

[35] Carlson J. (2024). Organizational Virtue Ethics and Moral Distress among Healthcare Workers. J. Clin. Ethics.

