# Supplementary material for: Moral Distress and Its Determinants among Nursing Students in an Italian University: A Cross-Sectional Study
Source: Nurs Rep. 2024 Aug 27;14(3):2140–52. doi: 10.3390/nursrep14030160 (PMC11417727; doi:10.3390/nursrep14030160)
Supplement: Supplementary file 1 [file nursrep-14-00160-s001.zip › nursrep-3158290-supplementary.pdf]

Supplementary Table S1. Moral Distress intensity in nursing students. SD: Standard Deviation; IQR: Interquartile Range; N: Total sample; n: number of subjects who had experienced a morally distressful situation described in the Question/Dimension. Only those situations with a reported frequency of at least 1 (indicating that the student has experienced the situation at least once) are considered in the analysis.

Dimensions	Questions (N=344)	Occurrence of the Event % and CI95%	Mean ± SD	Median; IQR (Min - Max)
D1	Q1. Identify plagiarism by a student (e.g. observing students copying during the exam, others' academic papers claimed as their own) (n = 302)	87.79% (91.25% - 84.33%)	2.5 ± 1.4	2; 2 (0 - 6)
D1	Q2. Perceive change of a fellow student's grades, by the teacher, to avoid failure (n = 216)	62.79% (67.9% - 57.68%)	2.06 ± 1.19	2; 2 (0 - 6)
D1	Q3. Witness inappropriate behaviors among students (e.g. no respect for rules regarding didactical activities in class or during clinical training, inadequacy in outfit/accessories in class and in clinical training, improper use of gloves, computer and drugs during clinical training). (n = 296)	86.05% (89.71% - 82.38%)	2.81 ± 1.61	2; 3 (0 - 6)
D3	Q4. Identify a mismatch between theoretical knowledge and practical application in one's teaching–learning process (n = 321)	93.31% (95.95% - 90.67%)	2.88 ± 1.54	3; 2 (1 - 6)
D2 and D3	Q5. Identify teachers' errors concerning content mastery (n = 292)	84.88% (88.67% - 81.1%)	2.34 ± 1.42	2; 2 (0 - 6)
D2	Q6. Perceive intimidating relations in the classroom (from other students/teachers) (n = 227)	65.99% (70.99% - 60.98%)	2.75 ± 1.58	2; 3 (0 - 6)
D3 and D4	Q7. Perceive divergences between the way clinical nurses and teachers perform procedures (n = 318)	92.44% (95.24% - 89.65%)	3.1 ± 1.54	3; 2 (1 - 6)
D2	Q8. Perceive discrimination by teachers (n = 212)	61.63% (66.77% - 56.49%)	2.6 ± 1.48	2; 2 (0 - 6)
D2	Q9. Perceive intimidating relations during practical activities (from other students, nurses, chief, physician) (n = 259)	75.29% (79.85% - 70.73%)	2.76 ± 1.54	2; 2 (0 - 6)
D4	Q10. Identify users' access difficulties to care (e.g. long waiting lists, no hospital beds, long waiting in emergency room) (n = 323)	93.9% (96.43% - 91.37%)	3.32 ± 1.58	3; 3 (0 - 6)
D4	Q11. Observe lack of continuity of user care during training activities (e.g. do not respond to a patient request, hygienic delayed or missing care) (n = 310)	90.12% (93.27% - 86.96%)	2.85 ± 1.56	2.5; 2 (0 - 6)
D4	Q12. Improvise to cope with lack of material in user care (n = 318)	92.44% (95.24% - 89.65%)	2.98 ± 1.54	3; 2 (1 - 6)
D5	Q13. Work with insufficiently prepared professionals to provide the necessary user care (n = 295)	85.76% (89.45% - 82.06%)	2.65 ± 1.47	2; 2 (0 - 6)

D5	Q14. Witness disrespect for users' rights by health students, nurses, support service and other health team professionals during training activities (n = 291)	84.59% (88.41% - 80.78%)	2.58 ± 1.55	2; 2 (0 - 6)
D5	Q15. Witness the commitment of user care due to bad/incomplete communication among the teams (n = 297)	86.34% (89.97% - 82.71%)	2.41 ± 1.37	2; 2 (0 - 6)
D5	Q16. Observe improper user care provided by students (n = 266)	77.33% (81.75% - 72.9%)	2.23 ± 1.38	2; 2 (0 - 6)
D5	Q17. Observe improper user care provided by health professionals (e.g. insert a bladder catheter to manage incontinence; leave the peripheral venous catheter, even if no longer necessary; make habitual use of restraint to prevent falls; perform pre-operative trichotomy with a razor blade; evaluate the pain without using standardized scales) (n = 300)	87.21% (90.74% - 83.68%)	2.65 ± 1.56	2; 2 (0 - 6)
D5	Q18. Observe inappropriate user care by family members (e.g. seeing family members feeding a patient who is not completely conscious) (n = 277)	80.52% (84.71% - 76.34%)	2.37 ± 1.39	2; 2 (0 - 6)
D5	Q19. Observe inappropriate information to the user (e.g. about therapeutic project, discharge) (n = 258)	75% (79.58% - 70.42%)	2.25 ± 1.38	2; 2 (0 - 6)
D5	Q20. Perceive the application of procedures to a user without consent (n = 193)	56.1% (61.35% - 50.86%)	2.35 ± 1.49	2; 2 (0 - 6)
D5	Q21. Observe situations of disrespect for the user's privacy (n = 192)	55.81% (61.06% - 50.57%)	2.35 ± 1.49	2; 2 (0 - 6)
D5	Q22. Observe a breach of confidentiality/professional secret of the user's personal information (n = 182)	52.91% (58.18% - 47.63%)	2.19 ± 1.38	2; 2 (1 - 6)
D5	Q23. Observe actions that compromise the user's dignity (e.g. undress the patient without using a screen, to violate the patient's shame, a relationship with the patient using the 2nd person instead of the 3rd) (n = 266)	77.33% (81.75% - 72.9%)	2.7 ± 1.65	2; 3 (0 - 6)
D5	Q24. Observe a violation of user safety (no patient identification before blood specimen collection, drugs administration and surgical procedures) (n = 238)	69.19% (74.07% - 64.31%)	2.46 ± 1.52	2; 2 (0 - 6)
D5	Q25. Perceive prejudice towards the users (n = 278)	80.81% (84.98% - 76.65%)	2.6 ± 1.57	2; 2.75 (0 - 6)
D5	Q26. Observe medical students performing procedures on users simply to improve their skills (n = 256)	74.42% (79.03% - 69.81%)	2.39 ± 1.54	2; 2 (0 - 6)
D5	Q27. Observe nursing students performing procedure on users merely to improve their skills (n = 279)	81.1% (85.24% - 76.97%)	2.61 ± 1.57	2; 2 (0 - 6)
D5	Q28. Perceive care provision to the user only aimed at postponing his death (e.g. therapeutic persistence) (n = 211)	61.34% (66.48% - 56.19%)	2.59 ± 1.56	2; 2 (0 - 6)
D5	Q29. Find oneself powerless when realizing a medication administration error (n = 239)	69.48% (74.34% - 64.61%)	2.43 ± 1.47	2; 2 (1 - 6)

D5	Q30. Experience delegation of nursing care to users' relatives (e.g. asking relatives to do hygienic care for nursing workload) (n = 234)	68.02% (72.95% - 63.09%)	2.19 ± 1.27	2; 2 (0 - 6)
D5	Q31. Observe compliance with team custom not to tell the truth to the user, even when the user asks for the truth (n = 246)	71.51% (76.28% - 66.74%)	2.45 ± 1.52	2; 2 (0 - 6)
**Dimensions**		**Occurrence***	**Mean ± SD**	**Median; IQR (Min - Max)**
D1. Disrespect for the ethical dimension of vocational training		78.88% (83.19% - 74.56%)	2.42 ± 1.19	2.33; 1.5 (0 - 6)
D2. Authoritarian teaching practice		71.95% (76.7% - 67.2%)	2.46 ± 1.28	2.13; 1.75 (0.5 - 6)
D3. Lack of competence of the teacher		90.21% (93.35% - 87.07%)	2.73 ± 1.29	2.5; 2 (0.67 - 6)
D4. Improper institutional condition to teach user care		92.22% (95.05% - 89.39%)	3.01 ± 1.35	2.75; 2 (0.33 - 6)
D5. Commitment of the ethical dimension of care		73.41% (78.08% - 68.74%)	2.34 ± 1.12	2.11; 1.6 (0.23 - 5.74)
Overall It-ESMEE score		76.82% (81.28% - 72.36%)	2.49 ± 1.06	2.29; 1.48 (0.68 - 5.65)

* At least point 1 as frequency of the specific situation.
